# Tackling human and animal health threats through innovative vaccinology in Africa

**DOI:** 10.12688/aasopenres.12877.2

**Published:** 2018-11-19

**Authors:** George M. Warimwe, Jyothi Purushotham, Brian D. Perry, Adrian V.S. Hill, Sarah C. Gilbert, Baptiste Dungu, Bryan Charleston

**Affiliations:** 1 Kenya Medical Research Institute-Wellcome Trust Research Programme, Kilifi, Kenya; 2 The Pirbright Institute, Woking, GU24 0NF, UK; 3 The Jenner Institute, University of Oxford, Oxford, OX3 7DQ, UK; 4 M.C.I. Santé Animale, Mohammedia, 28810, Morocco

**Keywords:** Vaccines, Africa, Capacity

## Abstract

Africa bears the brunt of many of the world’s most devastating human and animal infectious diseases, a good number of which have no licensed or effective vaccines available. The continent’s potential to generate novel interventions against these global health threats is however largely untapped. Strengthening Africa’s vaccine research and development (R&D) sector could accelerate discovery, development and deployment of effective countermeasures against locally prevalent infectious diseases, many of which are neglected and have the capacity to spread to new geographical settings. Here, we review Africa’s human and veterinary vaccine R&D sectors and identify key areas that should be prioritized for investment, and synergies that could be exploited from Africa’s veterinary vaccine industry, which is surprisingly strong and has close parallels with human vaccine R&D.

## Introduction

Vaccines are among the most cost-effective health interventions ever deployed. With the global eradication of two major infectious diseases and substantial reductions in the burden of several others attributed to their use ^1^, the impact of vaccines on global health is obvious. In the fish farming industry prophylactic vaccination has contributed to the near elimination of antimicrobial use in some settings ^2^, highlighting a potential role for vaccines in tackling the ongoing rise in infections caused by antimicrobial-resistant pathogen strains ^3^. However, safe and effective vaccines against many important human and animal diseases in Africa are lacking. Where vaccines are available, determining what aspect of vaccine deployment to prioritize for allocation of the scarce resources against many competing demands is often challenging partly due to inadequate vaccine policies. A strong African vaccine research and development (R&D) sector would accelerate the development of countermeasures against the most pressing, but currently neglected, local infectious disease needs. Furthermore, it would stimulate research into the immunoepidemiological factors that may have an impact on the performance and eventual deployment of candidate vaccines in Africa.

However, the development of a sustainable African vaccine R&D sector requires substantial long-term investment. Key areas for which greater funding is required are in increasing and strengthening the human resource capital in technical, institutional and policy areas, increasing the range, quality and technical capacity of vaccine development platforms, and augmenting the partnerships between public sector aspirations for vaccine-based disease control and private sector market opportunities. Sources of funding will include national governments, businesses, philanthropies, banks and international organizations ^4^. With the continent accounting for a dismal 0.5% of patent applications globally ^5^, we envisage that such investment would catalyze a product-driven scientific culture whilst incentivizing partnerships with established vaccine R&D enterprises within and outside Africa. To guide this approach, we contextualize here the typical vaccine R&D pipeline to Africa and identify key areas where focused investment can help accelerate discovery and development of novel interventions against local health priorities and emergencies.

The vaccine development process can be summarized as a multi-disciplinary pipeline, encompassing five broad stages that span: 1) definition of a target product profile to guide the vaccine R&D programme, informed by an understanding of the epidemiology and socioeconomic impact and priority of the intended disease target; 2) antigen / immunogen discovery; 3) formulation and testing in animal models; 4) biomanufacture and evaluation in the relevant target species; and 5) licensure and post-marketing evaluations, whilst taking account of the context in which the product is to be used (e.g. national vaccine policies, livestock production systems) to allow accurate estimation of vaccine impact.

### The demand for individual and institutional human resource capacity

As with any successful scientific research activity, appropriately skilled personnel are key to each stage of the vaccine R&D process. In addition, personnel need to be able to operate in an encouraging, supportive and technically well-endowed environment. Scientists are needed with the technical know-how to design, construct and evaluate candidate vaccines, supported by an enabling research environment and regulatory framework. The distribution of these human resource ingredients is highly skewed in Africa, and moreover the quality is variable. Nevertheless, there have been many capacity development programmes in the continent, with several major research programmes now providing high quality PhD and post-doctoral training in different biomedical and veterinary disciplines throughout Africa; however, few offer specific training in vaccinology.

For instance, the Wellcome Trust, in partnership with the Alliance for Accelerating Excellence in Science in Africa (AESA) ^6^ and other funders, awarded close to US$100 million to 11 research centers across Africa to support the development of African research leaders to tackle the continent’s health challenges. The UK Medical Research Council (MRC) and Department for International Development (DfID) have similarly invested in research centres in Africa, including a fellowship scheme – MRC/DfID African Research Leader scheme – dedicated to developing African research leaders. The Bill and Melinda Gates Foundation has invested heavily in human and animal health research programmes in Africa, further complementing capacity development programmes in these subject areas, such as the Biosciences eastern and central Africa-International Livestock Research Institute hub ( BecA-ILRI hub). More recently AESA has initiated the Coalition for African Research and Innovation ( CARI) with a vision to “build a highly coordinated, well-funded, and African-led African innovation enterprise”. This platform ultimately aims to catalyse and support innovation in Africa (including vaccines), with the CARI leadership group including African scientific leaders, major pharma executives and international funders.

The scientific base is therefore set to grow year on year providing a source of highly skilled individuals who can be guided towards a vaccine R&D career path within the continent and provided with appropriate mentorship if they decide to follow this course. Short – typically 1 week – vaccinology courses for African scientists are regularly run in Africa by institutions such as the Jenner Institute’s “ Vaccinology in Africa” and the University of Cape Town’s “ Vaccines for Africa” courses. The World Health Organization (WHO) Regional Office for Africa also organizes regular vaccinology courses within the continent, whilst courses based overseas, such as Fondation Mérieux’s two-week Advanced Course of Vaccinology ( ADVAC) and University of Siena’s Master in Vaccinology and Pharmaceutical Clinical Development, have dedicated scholarships for African scientists. These vaccinology courses tend to be highly oversubscribed, indicating strong interest in vaccinology within the continent. Transforming this interest into actual careers in vaccinology in Africa should be readily achievable for two main reasons.

First, the requisite laboratory consumables and infrastructure for early-stage vaccine design and development are no different to those required for other biomedical and veterinary research programmes already established at major research centres in Africa. Mobilizing existing resources within these institutes can, therefore, offset the initial capital investment required for early-stage vaccinology. Second, PhD trainees and post-docs in these research programmes are generally proficient in the application of generic skills in molecular biology, immunology, and statistical analysis for their research, all of which are essential for innovative vaccinology. Thus, joint doctoral and post-doctoral training programmes between African institutes and leading vaccine R&D centres globally are an obvious, feasible and attractive approach to building a career track in vaccinology in Africa. These provide long-term capacity in the design, development and evaluation of vaccines against human and animal diseases judged to be most important in the continent. A substantial limitation to achieving this has been the dearth of post-doc positions in African health science institutions. A major step change in this has been the funding by the Wellcome Trust of the African Institutions Initiative in 2009, in which seven new international and pan-African consortia were created. The partnerships - each led by an African institution - aimed to develop institutional capacity to support and conduct health-related research vital to enhancing people’s health, lives and livelihoods. An independent report on the impact of this funding has recently been published ^7^, which concluded that the initiative helped to lay the foundations for increased research capacity and the emergence of locally relevant health research agendas.

Building capacity of individual scientists has moved forward substantially, but is compromised in many countries by the inadequacies in institutional support to attract emerging graduates, with an inevitable lure to institutions in western countries where research facilities and funding are more available. Institutional strengthening in the African health sciences remains a major challenge ^6^. 

### Target diseases, vaccine profiles and partnerships

Vaccinology is clearly a demand driven science, and the demand in African settings comes in different forms. An assessment of the priority animal health research needs to contribute to processes of poverty reduction was carried out in 2002 ^8^, and this played an important role in defining funding priorities for several donor agencies, including the Bill and Melinda Gates Foundation. However, different donors have different priorities. For instance, the threat of emerging pandemic threats has influenced funding by the United States Agency for International Development (USAID) for the EPT2 programme ^9^. This focuses on helping countries detect viruses with pandemic potential, improve laboratory capacity to support surveillance, respond in an appropriate and timely manner, strengthen national and local response capacities, and educate at-risk populations on how to prevent exposure to these dangerous pathogens. The target countries in Africa are: Benin, Burkina Faso, Cameroon, Chad, Côte d’Ivoire, Egypt, Ethiopia, Ghana, Guinea, Kenya, Liberia, Mali, Niger, Nigeria, Tanzania, Uganda, Senegal, Sierra Leone, South Africa, Sudan and Togo ^9^.

At the country level demand for vaccine R&D and production should ideally be driven by the market, but in many countries the market assessments are rudimentary, and often driven more by available public sector funding than by sustainable development objectives ^10^. Priority setting is one thing; but ensuring the effective context of vaccine use is another essential ingredient of African vaccinology. This requires a combination of structured epidemiological studies, and an effective understanding of the roles of different stakeholders in technology delivery and uptake. This is effectively illustrated in studies on Rift Valley fever in eastern Africa ^11– 13^, and played a major role in the eradication of rinderpest ^14^.

### Antigen discovery

An increasing number of pathogen genome sequences are now available from open access repositories such as GenBank® and these have made *in silico* prediction of antigens with potential as candidate vaccines (also called reverse vaccinology) against any infectious disease possible wherever there is internet connectivity. This is not a limitation for African research institutes as internet connectivity is available in the continent. For example, one of the leading vaccine development programmes for East Coast fever, a devastating protozoan cattle disease that threatens livestock production in eastern Africa, is housed at the International Livestock Research Institute in Kenya and has a strong emphasis on *in silico* methodology for antigen discovery ^15, 16^.

Africa has exceptional capacity for empirical antigen discovery based on iterative longitudinal cohort studies in which naturally acquired immune responses to candidate antigens are measured at baseline and related to the risk of developing disease over a defined follow up period. Antigens targeted by immune responses shown to correlate with reduced odds of developing disease can then be prioritized for use in candidate vaccines, as is being done for malaria vaccines ^17^. However, this may not always be the best approach as it only works well for infections that elicit strong naturally acquired immunity. Where natural acquisition of immunity is poor reverse vaccinology can be useful in identifying antigens that are poorly immunogenic during natural infection, but highly potent when formulated into a vaccine ^18^. This is a leading vaccine development approach for life-threatening meningitis and septicaemia caused by serogroup B *Neisseria meningitidis* (Men B), and is the basis on which GlaxoSmithKline’s licensed Men B vaccine, Bexsero®, was developed ^18^. Further, empirical approaches based on the use of homologues of protective antigenic targets for vaccine development against different, but closely related, organisms have led to the world’s first licensed vaccine against porcine cysticercosis (Cysvax ^TM^, Indian Immunologicals Ltd) ^19^. The vaccine is licensed for use in pigs, in which vaccination is expected to interrupt transmission to, and impact on the incidence of, neurocysticercosis in humans ^19, 20^.

Excellent facilities and frameworks for epidemiological studies, including longstanding human demographic health surveillance systems and extensive livestock sample archives, exist in Africa and these have underpinned major projects identifying immune correlates of disease risk ^21^. Such well-curated sample archives require considerable investment and have been supported largely through long-term grant funding from international organizations such as the Wellcome Trust, UK Medical Research Council, Bill and Melinda Gates Foundation, among others. However, these costs are offset by the public health impact of the usually definitive studies they allow, including long-term trends in pathogen transmission, disease burden, effectiveness of interventions and prioritization of vaccine candidates or vaccine deployment based on circulating pathogen serotypes or strains.

Development of vaccines composed of live or inactivated whole organisms may obviate the need for extensive antigen discovery studies but determining the key targets of the protective immune response they elicit is often necessary to allow standardization of administration regimens and dosage. Further, for veterinary indications, whole organism vaccines do not allow differentiation of infected from vaccinated animals (so called ‘DIVA’) since the immune response elicited by vaccination cannot be distinguished from that elicited by natural infection. DIVA tests allow surveillance and vaccine deployment during animal disease outbreaks and are usually designed to detect immune responses to non-protective components absent from the vaccine construct but present in the whole target pathogen. DIVA compatibility is an important feature of target product profiles for animal vaccines, but the concept could also benefit human vaccinology. For instance, it is currently not possible to distinguish humans infected with tuberculosis from those immunised with the whole organism BCG vaccine on the basis of immune responses, though candidate subunit vaccines in development will address this in future ^22^. Nevertheless, irrespective of the type of vaccine candidate (subunit or whole organism), immunogen design and formulation and testing in animal models are arguably the rate-limiting steps to any sustainable vaccine R&D programme. This tends to be the stage at which the first go/no go decisions are made on which constructs to prioritize for further, increasingly costly, development. In addition, it is these data that underpin intellectual property applications. Whilst such pre-clinical testing is mostly done in mice, early stage assessments in natural host species of the disease indication provide rapid, more informative, host-pathogen systems to study vaccine performance ^23– 26^.

### Vaccine formulation and testing

Multiple platforms are available for candidate vaccine formulation, including DNA and RNA, protein-in-adjuvant, viral vectors, virus-like particles, inactivated whole organisms among others ^27, 28^. The design, formulation and production of vaccines utilising these platforms often involves bacterial, mammalian, plant or insect cell culture and expression systems that are readily available in Africa from global commercial suppliers. The technical know-how of formulating a promising antigen into a candidate vaccine against a particular disease indication can readily be transferred to Africa through joint PhD or post-doctoral programmes with global vaccine R&D centres. However, facilities for testing vaccine constructs in laboratory animals in Africa and the requisite regulatory framework for doing this are thin on the ground, and this impacts on vaccine R&D outputs. 

**Figure 1.**
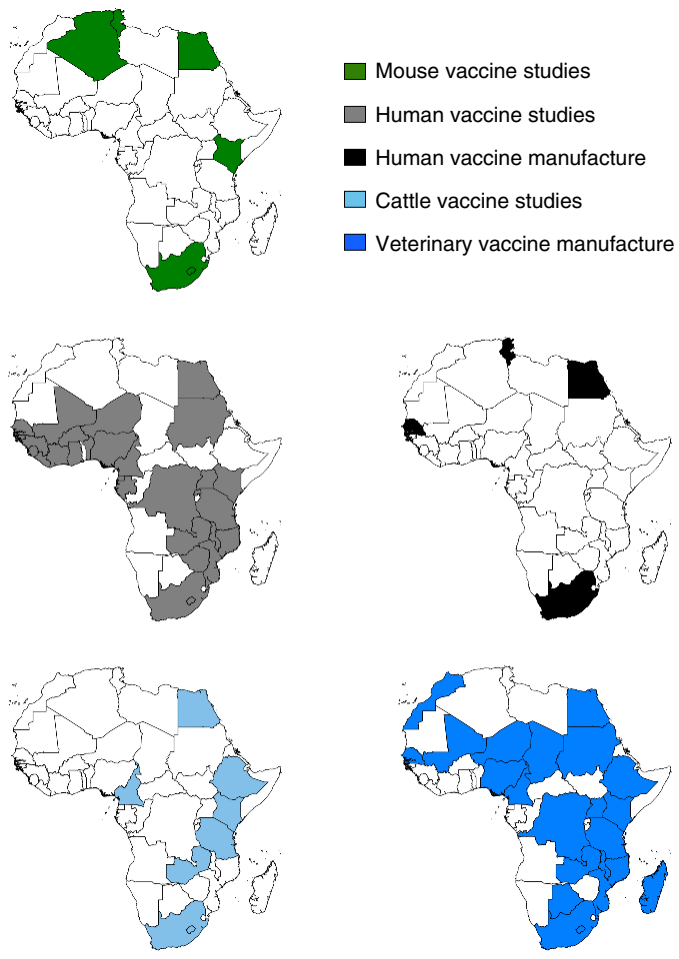
Vaccine research and development (R&D) and biomanufacturing capacity in Africa. African countries with an entry in PubMed between 1 ^st^ January 2013 and 31 ^st^ December 2017 for vaccine studies in mice (33 out of 9455 articles) or cattle (25 out of 267 articles) are shown in green and light blue, respectively. The search terms used were, 1) mice: (((mouse[title/abstract] OR mice[title/abstract]) OR murine[title/abstract]) AND (((vaccine[title/abstract] OR vaccination[title/abstract]) OR immunization[title/abstract]) OR immunisation[title/abstract])) AND (“2013/01/01”[PDAT] : “2017/31/12”[PDAT]); 2) cattle: (((cattle[title/abstract] OR cow[title/abstract]) OR bovine[title/abstract]) AND (((vaccine[title/abstract] OR vaccination[title/abstract]) OR immunization[title/abstract]) OR immunisation[title/abstract])) AND (“2013/01/01”[PDAT] : “2017/31/12”[PDAT]). African countries with an entry for phase I-IV clinical trials in ClinicalTrials.gov with a commencement date between 1 ^st^ Jan 2013 and 31 ^st^ December 2017 are shown in gray shading (111 out of 1511 registered studies). This excludes studies that have been suspended, withdrawn, terminated or those whose status is unknown. Countries with capacity for human or veterinary vaccine manufacture are shown in black and blue, respectively.

To illustrate this, we performed a literature search of peer-reviewed research articles on vaccine studies in mice, the most commonly used pre-clinical animal model in vaccinology, over a 5-year period on PubMed (date range 1 ^st^ Jan 2013 to 31 ^st^ December 2017). Of 9455 mouse vaccine publications in the study period only 33 (0.3%) were conducted in Africa, mainly in Egypt and South Africa ( Figure 1). In contrast, African vaccine studies in cattle, a major target species for veterinary vaccines, accounted for 9.4% of 267 cattle studies published in the same study period ( Figure 1). For human vaccines, we reviewed all entries of phase I-IV vaccine trials on ClinicalTrials.gov with a commencement date between 1 ^st^ Jan 2013 and 31 ^st^ December 2017; of 1511 studies, 111 (7.3%) were conducted in Africa and these were distributed across 28 countries ( Figure 1).

Together, these data highlight the poor representation of Africa in pre-clinical vaccinology, making this a key area for targeted investment. The extensive distribution of human vaccine studies in the continent is noteworthy and is a product of investment in human vaccine trial capacity by the European and Developing Countries Clinical Trials Partnership (EDCTP) and other funders. The cattle vaccine studies were less extensively distributed in the continent, being mainly done in Kenya, Ethiopia, South Africa, Tanzania and Egypt ( Figure 1). Nevertheless, rapid progress of major livestock vaccine programmes (e.g. Rift Valley Fever and Malignant Catarrhal Fever vaccines in East Africa) has been achieved with regulatory oversight from national veterinary ministries ^14, 25, 29^. Notably, the availability of highly experienced personnel within the continent allowed effective targeted deployment of rinderpest vaccines that led to the eradication of the disease ^14^.

Harmonised regulatory procedures for registration of new products for veterinary use are now in place in the East African Community, and plans for regulatory harmonisation are in progress for the Southern Africa Development Community (SADC), with support from the Global Alliance for Livestock Veterinary Medicines ( GALVmed) ^30^. For humans, the African Vaccine Regulatory Forum (AVAREF), composed of heads of national regulatory authorities and ethics committees from different countries in Africa, has been working towards harmonised regulatory systems for product approvals and clinical trials across Africa, which has helped improve product development timelines ^31^. Other initiatives, such as ZAZIBONA in the SADC, are working towards regional harmonisation for human medicines.

Clearly there is some ongoing investment in the clinical (animal and human) phases of vaccine R&D in Africa, including activities in regulatory affairs. We envisage that addressing the pre-clinical vaccine R&D capacity gap will stimulate development of new vaccine programmes that should utilise, help sustain and expand the existing human and veterinary trial capacity in Africa.

### Large-scale biomanufacture

Large-scale biomanufacture is a critical stage in the vaccine development pipeline that links all the early stage R&D to the actual deployment and use of the product in the intended target population. It is an extremely costly component of the pipeline, involving diverse vaccine production methods and extensive quality control and assurance processes, all of which contribute to the final cost of the vaccine ^32^. The method of vaccine production and its suitability for industrial scale-up is thus an important consideration when defining the target product profile at the outset of any vaccine R&D programme. Ready availability of sufficient amount of affordable vaccine against a particular health threat ultimately underpins its effective use in human and animal populations. Such ‘preparedness’ could be in the form of vaccine stockpiles, or in the availability of biomanufacturing processes able to rapidly meet the surge capacity needs of a sudden increase in disease incidence such as during outbreaks.

For example, an insufficient supply of currently licensed vaccines was a major contributing factor to the scale of the recent yellow fever virus (YFV) outbreaks in Angola and the Democratic Republic of Congo ^33^, and Brazil has resorted to using fractional doses of vaccine to increase supply for containment of an ongoing epidemic. These licensed YFV vaccines, one of which is biomanufactured by a World Health Organization (WHO) certified facility at the Institut Pasteur in Dakar, Senegal ^34^, confer long-lived immunity but rely on an egg-based biomanufacture process with relatively low capacity for rapid scale-up during outbreaks compared to other vaccine production processes (e.g. cell-culture systems). This situation is not unique to YFV vaccines; development of novel, scalable, vaccine platforms whose bulk production is readily achieved with low cost of goods continues to be one of the major challenges to human and veterinary vaccine manufacture globally ^27, 32^.

The Biovac Institute, a public-private partnership in South Africa, Vacsera in Egypt and Institut Pasteur de Tunis in Tunisia are the only other entities with significant activities in human vaccine biomanufacturing in Africa ( Figure 1). The limited human vaccine manufacturing capacity in the continent is partly attributable to the huge capital investment required for vaccine manufacture to WHO Good Manufacturing Practice (GMP) standards and the associated costs of maintaining the highly specialised infrastructure ^32^. However, an additional factor, arguably the most important, is the weak business case for products targeting disease indications in Africa, owing to the low-income status of most countries in the continent. Consequently, the bulk of human vaccines used in Africa are manufactured either by large multinational pharmaceutical companies such as GlaxoSmithKline and Sanofi Pasteur or companies in Asia. The African Vaccine Manufacturing Initiative ( AVMI), launched in 2010, aims to “promote the establishment of sustainable human manufacturing capacity in Africa” by advocating for vaccine manufacture in Africa, encouraging local partnerships between existing manufacturers, and attracting financial resources and skills, including capacity development, for vaccine manufacture in Africa. Recently, AVMI, with support from the United Nations Industrial Development Organization and the WHO, conducted an analytical assessment of Africa’s human vaccine manufacturing and procurement mechanisms ^35^. Their report from that study acknowledges the very limited human vaccine manufacturing capacity in Africa, outlining key areas for investment for sustainable, preferably regional, human vaccine manufacturing ^35^; barriers to entry include the high capital costs associated with the necessary infrastructure, high costs of retaining a highly-specialised workforce, and establishment of quality management systems to achieve and maintain compliance with GMP standards ^35^.

In contrast to human vaccine manufacture, at least 20 countries in Africa have an institution that manufactures veterinary vaccines, mainly catering for national or regional needs ( Figure 1). These manufacturers are predominantly state-owned, with managerial input from veterinary ministries to whom all the revenues go. A few are fully private, industrial-scale commercial entities (e.g. MCI Santé Animale in Morocco, Deltamune in South Africa) that also offer contract manufacturing as a service. Regardless of the business model, all these institutions almost exclusively produce and supply livestock and poultry vaccines to farmers, with none offering products for dogs, cats and other pet animals. Manufacturing quality assurance standards are set by the World Organization for Animal Health and the African Union-Pan African Veterinary Vaccine Centre ( AU-PANVAC), the main veterinary vaccine regulator in Africa. Supply of vaccines produced by African manufacturers to other markets is, as expected, subject to the regulatory requirements in these settings (e.g. regulatory approval by European Medicines Agency for vaccines destined for European markets). However, given previous instances of infectious livestock diseases spreading beyond Africa to the Arabian Peninsula and Europe ^36, 37^, investment in meeting the regulatory standards in these ‘emerging markets’ may significantly boost the vaccine market size for African manufacturers and provide a ready source of vaccines for disease outbreak control in these settings when the need arises.

## Conclusions

Africa has the potential and capacity for a thriving globally competitive vaccine R&D sector. Addressing the gaps and challenges highlighted here requires strong research leadership and targeted investment into the development of regional African centres of excellence in vaccinology. Such centres could readily be incorporated into existing world-renowned biomedical and veterinary research institutes in the continent, many of which have active collaborations and partnerships with leading vaccinology groups abroad. Investing in pre-clinical testing facilities would not only link ongoing epidemiological antigen discovery programmes in these institutes to proof of concept testing in animal models; it would also provide a new research career track and allow intellectual property protection for novel solutions emerging from the continent that could provide an additional revenue stream whilst promoting product-driven scientific research.

The regulatory framework for pre-clinical studies and work involving biotechnology-derived vaccines should be strengthened, as this underlies development of novel vaccine platforms and regimens. Cross talk between human and veterinary vaccine regulators should be encouraged, since the methods used in the design, formulation, testing and manufacture of human and veterinary vaccines are similar. For the case of zoonotic diseases, there are now major One Health programmes co-developing single vaccines for use in both humans and the animal reservoirs of infection ^25, 26, 38^. Vaccine regulators in Africa also need to develop approval policies and vaccine use protocols (including for investigational products) that can be used during disease outbreaks. This is now a key recommendation for regulators globally, given recent experience with the Ebola disease epidemic in West Africa ^39, 40^. 

Developing a sustainable financing mechanism to incentivize new vaccine programmes in Africa will remain a big challenge, and the specific funding gaps will vary from region to region given geographic variations in disease burden ^4, 35^. However, in recognizing this funding gap there are now new international initiatives focused on supporting research and development of countermeasures against emerging infectious diseases in Africa. These include the WHO R&D blueprint, the UK Vaccine R&D Network, the Coalition for Epidemic Preparedness Innovations ( CEPI), and more recently, the Coalition for African Research and Innovation ( CARI) led by AESA. These initiatives offer a timely opportunity for African research institutes to participate in vaccine R&D. However, without the enabling infrastructure for pre-clinical testing of candidate products, Africa is very unlikely to take a lead role in providing novel vaccine solutions for global health threats such as Ebola, Lassa fever, Crimean-Congo haemorrhagic fever and other diseases that are endemic to the continent (and prioritized by WHO for urgent vaccine R&D). Having this in place will encourage product development partnerships with major manufacturers and accelerate development of effective interventions against the most pressing and neglected animal and human health needs.

## Disclaimer

The views expressed in this article are those of the author(s). Publication in AAS Open Research does not imply endorsement by the AAS.

## Data availability

All data underlying the results are available as part of the article and no additional source data are required.
