# Supplementary material for: Tackling human and animal health threats through innovative vaccinology in Africa
Source: AAS Open Res. 2018 Nov 19;1:18. Originally published 2018 Jul 2. [Version 2] doi: 10.12688/aasopenres.12877.2 (PMC7118973; doi:10.12688/aasopenres.12877.2)
Supplement: Peer review history

# Peer review: Reviewer response for version 2

**DOI:** 10.21956/aasopenres.13999.r26650

**Published:** 2018-11-19

**Authors:** Victor Mbao

**Affiliations:** 1 International Development Research Centre (IDRC), Nairobi, Kenya

Approved.

I confirm that I have read this submission and believe that I have an appropriate level of expertise to confirm that it is of an acceptable scientific standard.

# Peer review: Reviewer response for version 1

**DOI:** 10.21956/aasopenres.13943.r26507

**Published:** 2018-07-24

**Authors:** Allan Saul

**Affiliations:** 1 GSK Vaccines Institute for Global Health (GVGH), Siena, Italy

This article addresses the need for the development of vaccines to address heath problems in Africa and the challenges facing  their production in Africa.  It provides a realistic situation of the R&D environment and a balanced view of areas that have been progressing.  It makes an interesting contrast between the human and veterinary vaccine development.  This review is primarily written from the perspective of discovery and early clinical development  in the vaccine development path, including the need to train African Researchers.

 It does briefly highlight some of the issues in taking a vaccine from proof of concept to registration, manufacture and delivery but really big challenges include building the investment case to balance future sales against costs of  registration trials and the capital expenditure required for  manufacture as well as subsequent ongoing production costs.  Despite development such as AVMI and AVAREF there is still major gaps in the ability of National Regulatory Authorities that could register a vaccine that could achieve WHO prequalification. Could there be another review, perhaps involving additional authors from African Vaccine Manufacturers that addresses these issues more fully?

 The drafting of the paper is high quality.  I did not find any minor issues that needed attention.

I confirm that I have read this submission and believe that I have an appropriate level of expertise to confirm that it is of an acceptable scientific standard.

# Peer review: Reviewer response for version 1

**DOI:** 10.21956/aasopenres.13943.r26505

**Published:** 2018-07-20

**Authors:** Victor Mbao

**Affiliations:** 1 International Development Research Centre (IDRC), Nairobi, Kenya

**Summary**

 The article clearly articulates the great potential for Africa to lead in vaccine R&D based on available skilled human capital, infrastructure and access to cutting edge technology (but for a few obstacles). It compares and contrast these aspects with ample evidence and examples. The challenges and strengths African vaccinology faces are presented in great detail, and some suggestions are proffered for their resolution. It logically tackles areas of human capacity, target product profiles, antigen discovery, formulation testing and bioprocessing. It concludes pointing to the African science potential, the need for smart funding mechanisms to stimulate and sustain the vaccinology field, the importance of strategic partnerships and the need to strengthen regulatory frameworks that govern clinical studies on biotechnologically derived vaccines. 

**My comments**

 This is a contemporary and very relevant article and brings to the fore a discussion that provides evidence for a vibrant vaccinology leadership by Africans for Africa. The comparison between human and veterinary vaccines gives a rich eye-opening reading in terms of the potential gains from adopting One-Health approaches to vaccine research. In my opinion, it would even be made stronger with consideration for the following:

**1. **Around the statement **"greater funding is required are in increasing and strengthening the human resource capital"** the incessant brain drain could have been more deeply addressed as it adversely affects efforts on capacitating African vaccinology. A few more suggestions on factors that could reverse the trend would have been helpful.

 2. In stating a few examples of international organisations that have invested in capacity building of African scientists, one example that should have been included is the International Development Research Centre (IDRC) of Canada which has been doing this for longer than some of the examples given. IDRC is currently funding 15 veterinary vaccine development projects in African and Asia.

 3. Infrastructure - This is a costly capital expenditure that limits much of the desired research as rightly pointed out by the authors. Equipment maintenance and calibration are significant challenges that need a systematic approach to effectively address. The authors could have used this opportunity to discuss this and offer some innovative solutions e.g. a shift towards procurement plans and contracts that include extended after sales service and spares?

 4. Political economies - In Africa as elsewhere, the research agenda does not escape political effects. How can we leverage for instance national science granting councils? Technical centers of continental or regional bodies? The authors do touch on regional economic communities and their enabling environment contribution to harmonised regulatory procedures. I think more discussion would have added value to showcasing the good that stands to be gained from smart navigation of the political environment in Africa.

I confirm that I have read this submission and believe that I have an appropriate level of expertise to confirm that it is of an acceptable scientific standard.
